# The role of STAT3/VAV3 in glucolipid metabolism during the development of HFD-induced MAFLD

**DOI:** 10.7150/ijbs.86465

**Published:** 2024-03-11

**Authors:** Yue Jiang, Pengcheng Luo, Yu Cao, Dewei Peng, Shengqi Huo, Junyi Guo, Moran Wang, Wei Shi, Cuntai Zhang, Sheng Li, Li Lin, Jiagao Lv

**Affiliations:** 1 Division of Cardiology, Department of Internal Medicine, Tongji Hospital, Tongji Medical College, Huazhong University of Science and Technology, Wuhan, China.; 2 Departments of Geriatrics, Tongji Hospital, Tongji Medical College, Huazhong University of Science and Technology, Wuhan, China.

**Keywords:** MAFLD, glucolipid metabolism, STAT3, VAV3, GLUT4

## Abstract

Metabolic-associated fatty liver disease (MAFLD) is a globally prevalent chronic hepatic disease. Previous studies have indicated that the activation of the signal transducer and activator of transcription3 (STAT3) plays a vital role in MAFLD progression at the very beginning. However, the specific association between STAT3 and abnormal hepatic metabolism remains unclear. In this study, activated inflammation was observed to induce abnormal glucolipid metabolic disorders in the hepatic tissues of high-fat diet (HFD)-fed ApoE^-/-^ mice. Furthermore, we found that the activation of STAT3 induced by HFD might function as a transcriptional factor to suppress the expression of VAV3, which might participate in intracellular glucolipid metabolism and the regulation of glucose transporter 4 (GLUT4) storage vesicle traffic in the development of MAFLD both *in vitro* and *in vivo*. We verified that VAV3 deficiency could retard the GLUT4 membrane translocation and impair the glucose homeostasis. Additionally, VAV3 participates in cholesterol metabolism in hepatocytes, eventually resulting in the accumulation of intracellular cholesterol. Moreover, rAAV8-TBG-VAV3 was conducted to restore the expression of VAV3 in HFD-fed ApoE^-/-^ mice. VAV3 overexpression was observed to improve glucose homeostasis as well as attenuate hepatic cholesterol accumulation *in vivo*. In conclusion, the STAT3/VAV3 signaling pathway might play a significant role in MAFLD by regulating glucose and cholesterol metabolism, and VAV3 might be a potential therapeutic strategy which could consequently ameliorate MAFLD.

## Introduction

Metabolic-associated fatty liver disease (MAFLD), also known as non-alcoholic fatty liver disease (NAFLD), is a multi-factor induced chronic hepatic disease 1 that affects over a quarter of the world's adult population 2, 3. It is defined as the presence of hepatic steatosis with obesity, type 2 diabetes mellitus (T2DM), or metabolic abnormalities 4 and is considered an independent risk factor for many severe chronic diseases such as atherosclerosis, hypertension, and hepatoma 5-8. The number of patients with MAFLD increases yearly, resulting in a tremendous financial burden on national health care. However, currently, there is no approved therapeutic strategy for MAFLD 3.

Previous studies have indicated that STAT3 is activated in the liver during MAFLD development because systemic low-grade chronic inflammation is triggered 9. Once STAT3 is activated, it phosphorylates (P-STAT3), translocates to the nucleus through dimerization of P-STAT3, and acts as a transcription factor to modulate gene expression 10. Glucolipid metabolic disorders in MAFLD was observed, which might be due to the activation of STAT3; however, a detailed explanation is lacking 11, 12. In our study, the activation of STAT3 induced by HFD was verified. Moreover, the blood glucose abnormalities were detected, possibly due to abnormal GLUT4 membrane translocation in HFD-fed ApoE^-/-^ mice. GLUT4 is a key protein of 14 members of the GLUTs family regulated by insulin, maintaining systemic glucose homeostasis 13. It is mostly stored in the GLUT4 storage vesicles (GSVs) in the cytosol 14, 15. When stimulated by insulin, it rapidly responds to recruitment and translocates to the cell surface for glucose absorption. However, whether and how activated STAT3 contributes to abnormal GLUT4 traffic requires further investigation.

Based on the ChIP-seq results of hepatic tissues from HFD-fed ApoE^-/-^ mice, the focus was placed on a specific guanine nucleotide exchange factor (GEF) named VAV3, which belongs to the VAV family. As a GTPase regulator, VAV3 activates the Rho GTPase family in cancer and in the vascular endothelial barrier, and is potentially associated with metabolic syndrome and obesity 16-20. Activated GTPases such as Rac-1 and RhoA have also been reported to participate in the regulation of GSV traffic through dynamic actin remodeling 15, 21, 22. Therefore, activated STAT3 was speculated to manage GLUT4 translocation by regulating VAV3, but the specific mechanism requires further investigation. Since glucose and lipid metabolism can interact with each other, we also focused on lipid metabolism during the development of MAFLD 23. Once glucose metabolic disorders exist, they can also impact lipid metabolism; therefore, other lipid or cholesterol metabolic abnormalities may be associated with MAFLD. When it comes to cholesterol intracellular accumulation in hepatocytes, cholesterol synthetases that play vital roles, such as 3β-hydroxysterol-Δ24 reductase (Dhcr24), enzyme 7-dehydrocholesterol reductase (Dhcr7), and sterol 14 alpha-demethylase (Cyp51), increase 24, 25 while the reverse cholesterol transporter ATP-binding cassette transporter A1 (ABCA1) decreases, implying more cholesterol synthesis but less efflux 26, 27. In our study, VAV3 deficiency was observed to be related to intracellular cholesterol accumulation in HFD-induced hepatocytes.

In summary, the potential regulation of STAT3/VAV3 on the traffic of GLUT4 and hepatic cholesterol metabolism in the development of MAFLD was observed. We also indicated that recovering the expression of VAV3 under HFD could be a potential strategy for treating MAFLD and alleviating glucolipid metabolism and hepatic inflammation.

## Results

### HFD impaired the glucolipid metabolism and induced chronic inflammation in the development of MAFLD

**Figure 1:**
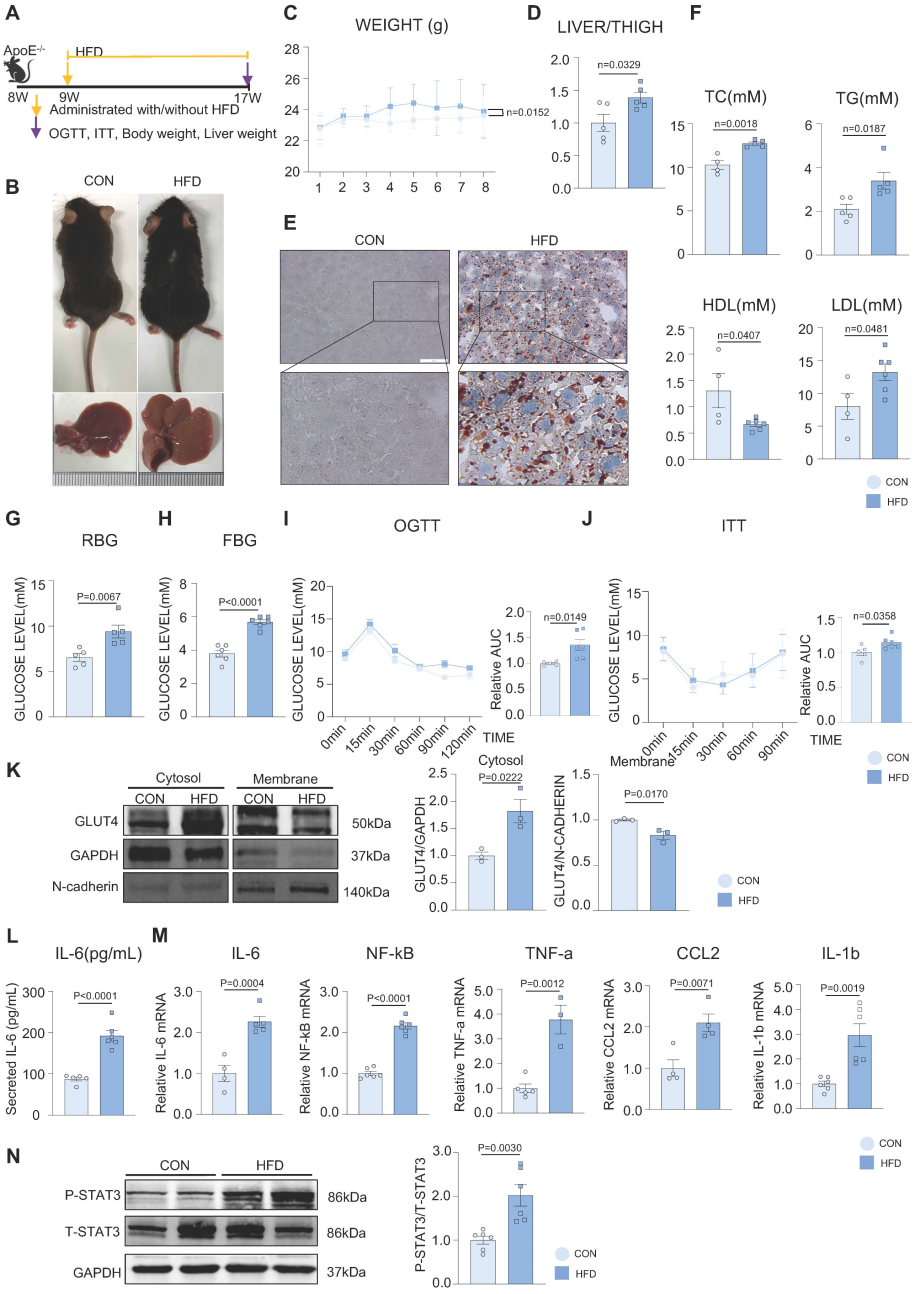
** HFD impaired the glucolipid metabolism and induced chronic inflammation in the development of MAFLD.** (A) Schematic illustration of the HFD-induced MAFLD mouse model. (B) Representative images of mouse and its liver isolated from mice. (C) Statistic analysis showed mouse body weight. (D) Statistic analysis demonstrated the liver weight/tibia length ratio. (E) Representative images of oil red O staining from mouse liver. (F) Statistic analysis demonstrated the serum levels of TC, TG, LDL and HDL. (G-J) Statistic analysis demonstrated random and fasting blood glucose levels and OGTT and ITT results. (K) Cytosol-membrane extracting demonstrated the GLUT4 distribution on membrane and cytosol in primary hepatocytes. (L) Statistic analysis demonstrated the serum IL-6 detected by ELISA. (M) qPCR showed mRNA levels of hepatic inflammatory cytokines IL-6, NF-kB, TNF-a, CCL2 and IL-1b. (N) Western blot showed the expression of P-STAT3. All data represent the means ± SEM; t-test.

Figure 1A represents an HFD-induced MAFLD model. After 8 weeks of HFD feeding, the fur of mice in the HFD group gradually became greasier, and body weight was elevated compared with mice in the control (CON) group (Figure 1B-C). The liver volume and liver weight/tibia length ratio were significantly higher in the HFD group than in the CON group (Figure 1B, 1D). Hepatic steatosis induced by HFD was verified by Oil red O staining, with more lipid droplets accumulating in the HFD group than in the CON group (Figure 1E). Additionally, the levels of serum total cholesterol (TC), total triglycerides (TG), and low-density lipoprotein (LDL) were significantly elevated, whereas those of high-density lipoprotein (HDL) were significantly decreased in the HFD group. (Figure 1F). Both the random and fasting blood glucose levels of the HFD group were elevated compared to those of the CON group (Figure 1G-H). We conducted OGTT and ITT tests to further investigate glucose metabolism in mice, which showed that HFD induced abnormal glucose tolerance and insulin inertia (Figure 1I-J). Furthermore, we found that the expression of phosphorylated IRS, IR, AKT (P-IRS, P-IR, P-AKT), and total GLUT4 was not different from that in the CON group, indicating that the canonical insulin pathway in the liver tissue remained unchanged in this phase (Supplementary Figure 1A). To further explore the glucose abnormality in MAFLD, a cytosol-membrane separation assay was conducted to detect GLUT4 intracellular translocation. GLUT4 was delayed in the cytosol rather than being transported to the membrane in mouse primary hepatocytes in the HFD group, suggesting that HFD caused defects in GLUT4 membrane traffic (Figure 1K). In addition, the mRNA levels of activated inflammatory cytokines, such as IL-6, NF-kB, TNF-a, CCL2, and IL-1b, were increased in the mouse liver and the secretion level of IL-6 was elevated in serum in the HFD group (Figure 1L-M). Besides, according to previous studies, IL-6 can activate STAT3 to phosphorylate (P-STAT3), and the IL-6/STAT3 signaling pathway is aberrantly hyperactivated in many chronic inflammatory diseases 28. In our study, STAT3 was also activated in the liver tissue of the HFD group (Figure 1N). These results suggested that hepatic inflammation might be activated during MAFLD development.

### Ox-LDL induced the activation of STAT3 in the development of MAFLD, resulting in defective GLUT4 traffic and cholesterol accumulation *in vitro*

**Figure 2:**
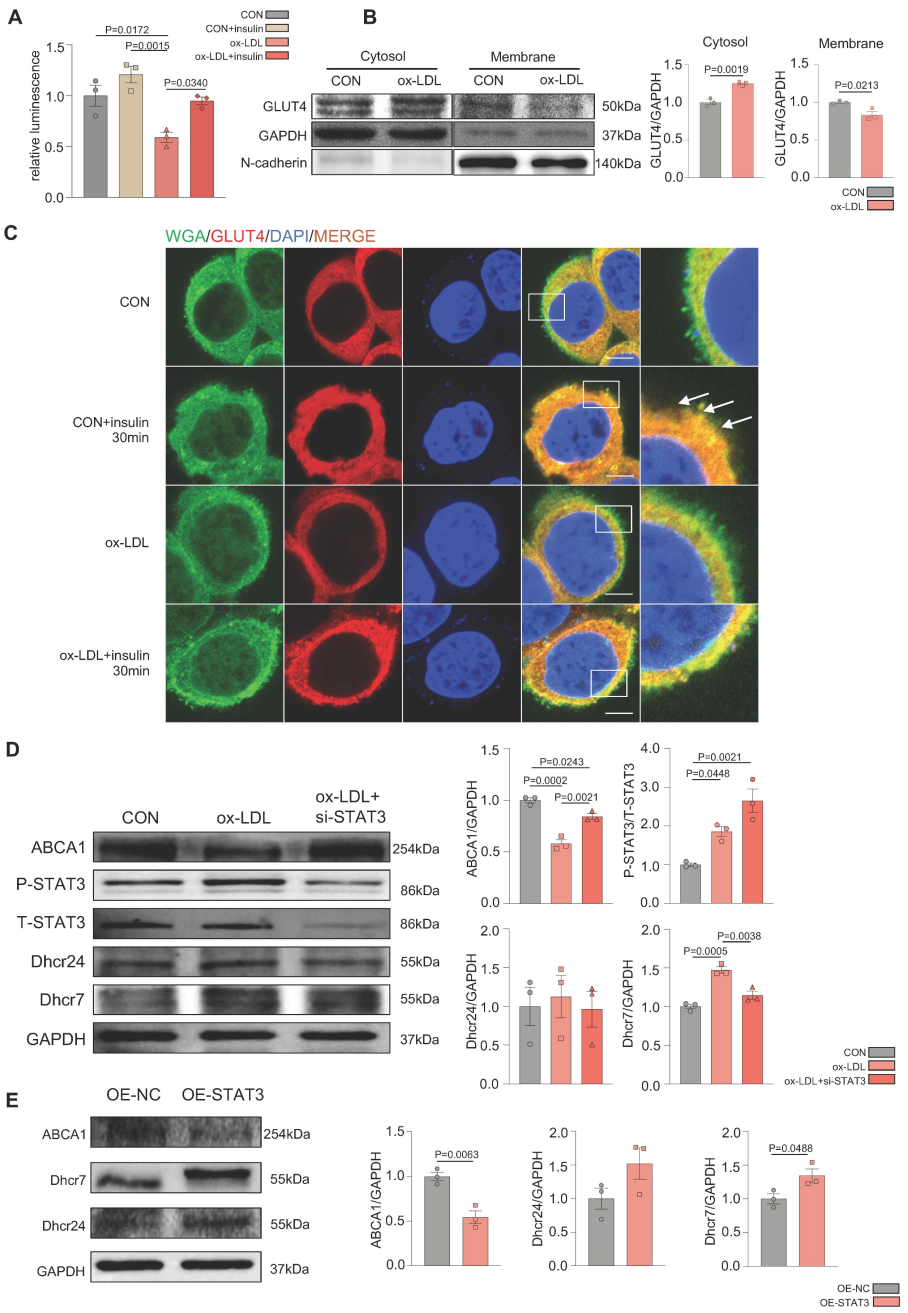
** Ox-LDL induced the activation of STAT3 in the development of MAFLD, resulting in GLUT4 traffic defect and cholesterol accumulation *in vitro*.** (A) Luminescence showed the glucose uptake level of L02 cells detected by 2-DG glucose uptake assay. (B) Cytosol-membrane extracting demonstrated the GLUT4 distribution on membrane and cytosol in L02 cells. (C) Representative images of immunofluorescence showed the colocation of membrane marker WGA (FITC-Green) and GLUT4 (Cy3-Red) Blue represents nuclear DNA staining by DAPI; green represents WGA staining; red represents GLUT4 staining. (D-E) Western blot showed the expressions of ABCA1, P-STAT3, Dhcr7 and Dhcr24 under ox-LDL with si-STAT3 or overexpression of STAT3.Data represent means ± SEM; t-test; One-way ANOVA.

To further demonstrate the glucolipid metabolic disorders in MAFLD *in vitro*, ox-LDL was used to establish an MAFLD model. Oil red O staining indicated that ox-LDL induced the accumulation of intracellular lipid droplets in HepG2 and L02 cells (Supplementary Figure 2A). The 2-deoxyglucose (2-DG) assay demonstrated that hepatic glucose uptake was significantly reduced in the ox-LDL group with ou without insulin, which suggested that ox-LDL induced hepatic glucose abnormality (Figure 2A). Interestingly, the expression of P-IRS, P-IR, P-AKT, and GLUT4 was not different between the two groups, indicating that the canonical insulin pathway remained unchanged (Supplemental Figure 2B). To determine the reason for the glucose metabolic disorders, the membrane and cytosolic distribution of GLUT4 was detected. The plasma membrane separation assay suggested that ox-LDL suppressed insulin-induced GLUT4 membrane translocation, with more GLUT4 in the cytosol and less in the membrane than that in the CON group (Figure 2B). Immunofluorescence results showed the co-localization of GLUT4 and the membrane marker WGA, also indicating a deficiency in GLUT4 membrane translocation (Figure 2C). Increased levels of the cholesterol synthetases Dhcr24 and Dhcr7 in the ox-LDL group were observed by western blotting, indicating that hepatic intracellular cholesterol synthesis was elevated 24, 25. Nevertheless, the expression level of reverse cholesterol transporter ABCA1 decreased, implying reduced cholesterol efflux 26. This suggests that cholesterol accumulation in hepatocytes was induced by ox-LDL (Figure 2D). Additionally, both IL-6 secretion and mRNA were significantly increased in L02 cells stimulated by ox-LDL, and the activation of STAT3 in L02 cells was observed (Supplemental Figure 2B-D). IL-6 was used to activate STAT3 in L02 cells and activation of STAT3 was detected, which induced impaired glucose uptake and abnormal GLUT4 membrane translocation, similar to that in the ox-LDL group (Supplemental Figure 2E-G). To further verify whether the stimulation of ox-LDL-induced hepatic cholesterol disorders was due to the activation of STAT3, we used small interfering RNA (siRNA) to knock down the expression of STAT3; resulting in relieved cholesterol accumulation (Figure 2D), while the overexpression of the STAT3 plasmid aggravated excessive cholesterol accumulation (Figure 2E).

### VAV3 might participate in the development of MAFLD regulated by STAT3

**Figure 3:**
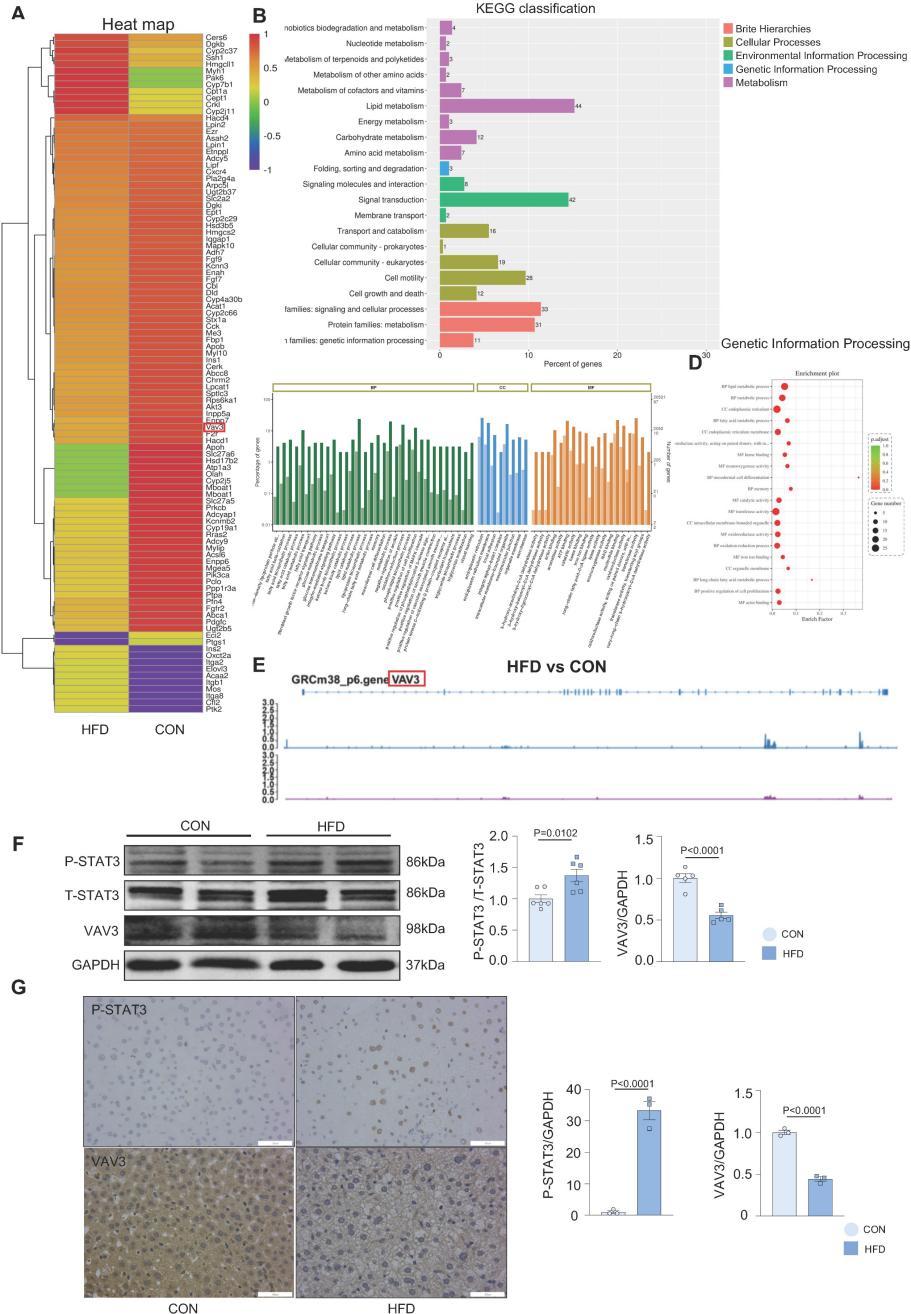
** VAV3 might participate in the development of MAFLD regulated by STAT3.** (A-D) The GO and KEGG enrichment analysis of STAT3 target genes according to ChIP-seq. STAT3 direct target genes in hepatic tissues were identified by ChIP-seq and subjected to GO and KEGG enrichment analysis. (E) Representative ChIP-seq peak located on STAT3 which directly targeted on gene VAV3. (F) Western blot showed the expressions of P-STAT3 and VAV3. (G) Representative images of P-STAT3 and VAV3 immunohistochemistry staining from hepatic tissues and were plotted as histograms through statistical analysis. All data represent the means ± SEM; t-test.

Overactivated STAT3 was verified to cause hepatic glucolipid metabolic disorders, such as GLUT4 membrane traffic deficiency and cholesterol accumulation in MAFLD. Considering that STAT3 is a transcriptional factor, we conducted ChIP-seq in liver tissues to probe genes transcriptionally regulated by STAT3 that could participate in regulating hepatic glucolipid metabolism. A visual analysis was performed and 63 genes, focused on glucolipid metabolism and regulation of GLUT4 storage vesicles, were screened (Figure 3A). According to the results of ChIP-seq and the prediction from the JASPAR database (Supplemental Figure 3A and Supplementary Table 1A), VAV3 was found to be regulated by STAT3 and as per the GO and KEGG databases, was associated with metabolism as a GEF protein that could activate Rho GTPases to regulate GLUT4 traffic 29; Figure 3B-D). As demonstrated by the annotated peaks of VAV3, there was potential enrichment of STAT3 binding to its sequence, suggesting that VAV3 could be transcriptionally regulated by STAT3 (Figure 3E). The decreased expression of VAV3 in hepatic tissues and the elevated P-STAT3 in the HFD group were verified by western blotting and IHC, in accordance with the ChIP-seq and JASPAR databases (Figure 3F-G). These results indicated that VAV3 might be regulated by STAT3 and participate in GLUT4 translocation, promoting MAFLD.

### Over-activation of STAT3 could suppress the expression of VAV3

**Figure 4:**
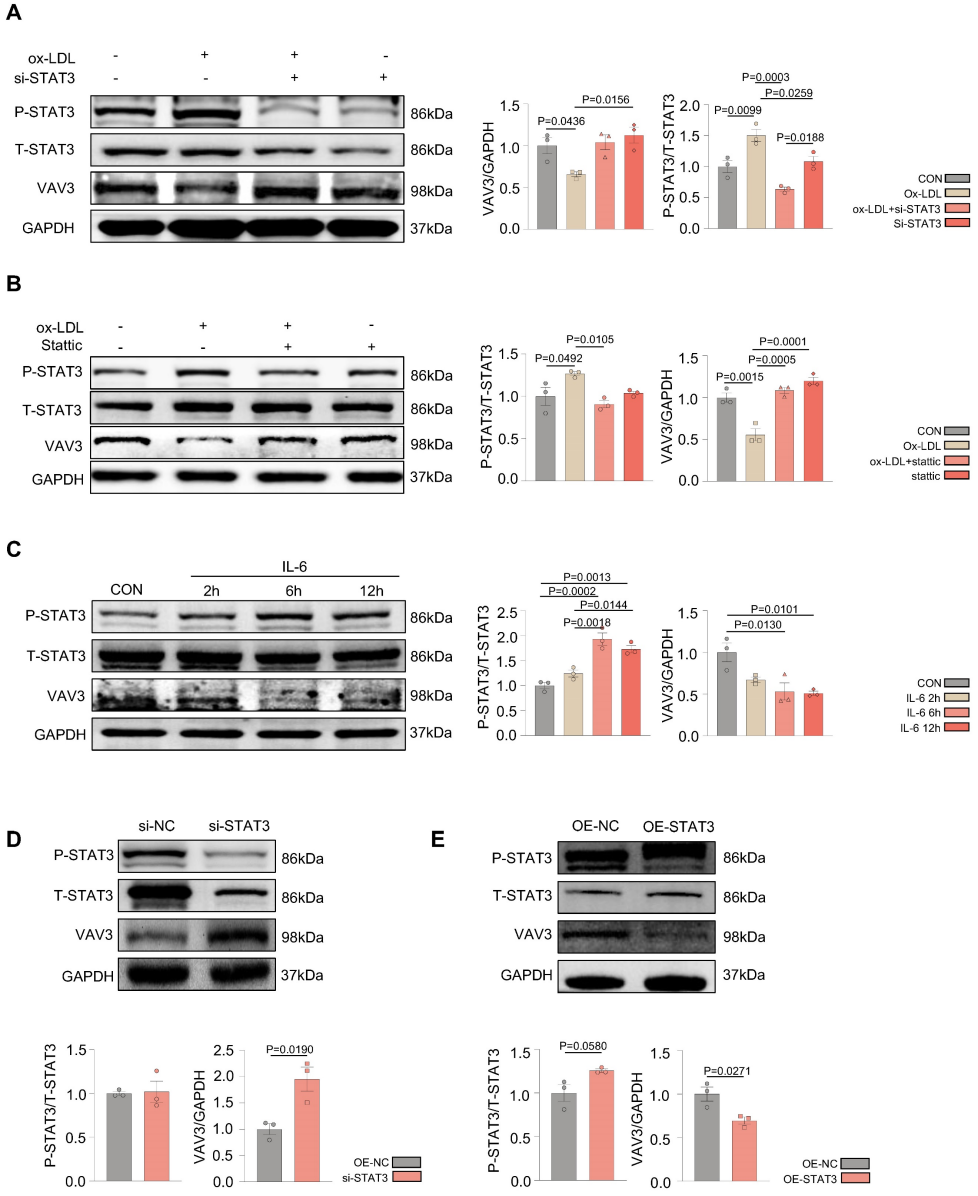
** Over-activation of STAT3 could suppress the expression of VAV3.** (A-B) Western blot demonstrated the expressions of P-STAT3 and VAV3 in L02 cells under ox-LDL stimulation with si-STAT3 or stattic. (C) Western blot demonstrated the expressions of P-STAT3, VAV3 and GLUT4 in L02 cells under IL-6 time-dependent manner 2h, 6h and 12h. (D-E) Western blot demonstrated the expressions of P-STAT3 and VAV3 in L02 cells under si-STAT3 or STAT3 overexpression. Data represent means ± SEM; t-test; One-way ANOVA.

According to the ChIP-seq analysis, to further probe whether STAT3 could regulate the development of MAFLD by suppressing the expression of VAV3, siRNA to knock down STAT3 as well as a specific phosphorylated STAT3 inhibitor stattic were used to intervene in L02 cells after ox-LDL stimulation. Ox-LDL induced decreased VAV3; however, when P-STAT3 was reduced, VAV3 expression increased accordingly (Figure 4A-B). In addition, IL-6 was used to activate STAT3 in both L02 cell lines and primary hepatocytes. The expression of VAV3 decreased while P-STAT3 was elevated in a time-dependent manner of IL-6 stimulation (Figure 4C, Supplemental Figure 4E). Furthermore, a siRNA to knockdown STAT3 and a plasmid to overexpress STAT3 were used to verify the negative regulation of STAT3 to VAV3 (Figure 4D-E). The expression of GLUT4, P-IRS, P-IR, and P-AKT stimulated by IL-6 or stattic were detected and STAT3 hyperactivation was found to have no impact on the canonical insulin signaling pathway at this phase, only impairing GLUT4 membrane translocation (Supplemental Figure 4A-D).

### VAV3 deficiency could induce defective GLUT4 translocation and cholesterol accumulation

**Figure 5:**
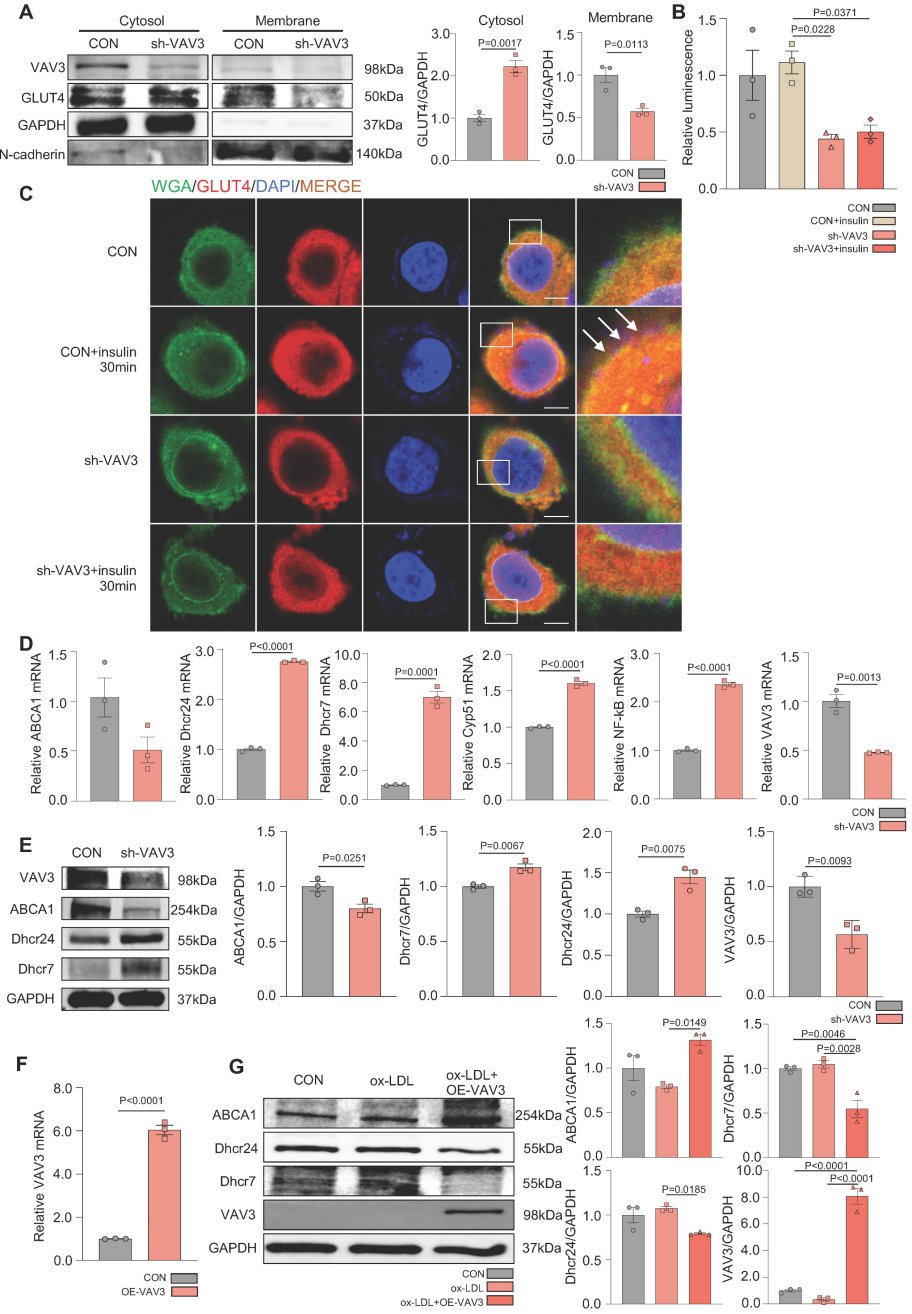
** VAV3 deficiency could induce defect of GLUT4 translocation and cholesterol accumulation.** (A) Cytosol-membrane extracting demonstrated the GLUT4 distribution on membrane and cytosol in L02 cells under shVAV3. (B) Luminescence showed the glucose uptake level of L02 cells detected by 2-DG glucose uptake assay. (C) Representative images of immunofluorescence showed the colocation of membrane marker WGA (FITC-Green) and GLUT4 (Cy3-Red). Blue represents nuclear DNA staining by DAPI; green represents WGA staining; red represents GLUT4 staining. (D) qPCR indicated the mRNA expressions of ABCA1, Dhcr24, Dhcr7, Cyp51, NF-kB and VAV3 in shVAV3 L02 cells. (E) Western blot showed the expressions of VAV3, ABCA1, Dhcr7 and Dhcr24 in shVAV3 L02 cells. (F) qPCR indicated the mRNA level of VAV3. (G) Western blot indicated the expressions of ABCA1, Dhcr24, Dhcr7 and VAV3 under ox-LDL with VAV3 overexpression. Data represent means ± SEM; t-test; One-way ANOVA.

To determine whether VAV3 could play a key role in regulating glucose and cholesterol accumulation, siRNA and shRNA lentiviral vector were used to knock down VAV3 and detected the VAV3 knockdown efficiency by qPCR and western blotting (Supplemental Figure 5A-D). Defective GLUT4 membrane translocation induced by VAV3 deficiency was then observed (Figure 5A-C and Supplemental Figure 5E). The expression of cholesterol synthetases Dhcr24, Dhcr7, and Cyp51 was elevated, whereas that of the reverse cholesterol transporter ABCA1 was decreased, indicating that intracellular cholesterol accumulation was caused by hepatic VAV3 deficiency (Figure 5D-E). Interestingly, VAV3 deficiency also increased the expression of NF-kB, which might cause other cellular impairments and require further exploration (Figure 5D). Nevertheless, adopting the VAV3 overexpression plasmid under ox-LDL stimulation alleviated intracellular cholesterol accumulation (Figure 5F-G).

### Recovering the expression of VAV3 could attenuate the development of MAFLD *in vivo*

**Figure 6:**
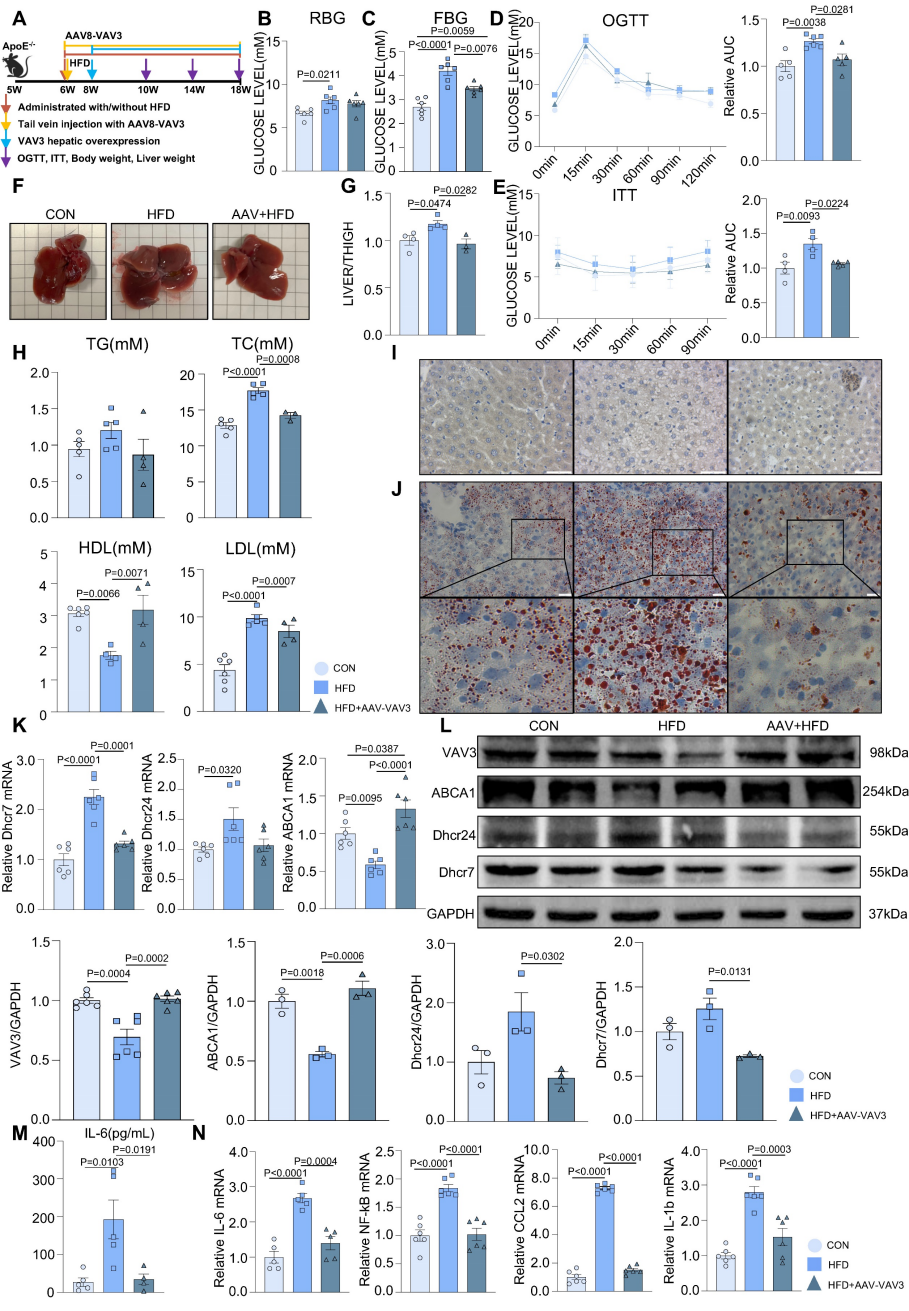
** Recovering the expression of VAV3 could attenuate the development of MAFLD *in vivo*.** (A) Schematic illustration of the HFD-induced MAFLD mouse model and rAAV8-TBG-VAV3 tail vein injection. (B-E) Statistic analysis demonstrated the random blood glucose, fasting blood glucose, OGTT and ITT test results. (F) Representative images of liver isolated from mice. (G) Statistic analysis demonstrated the liver weight/tibia length ratio. (H) Statistic analysis demonstrated the serum levels of TC, TG, LDL and HDL. (I) Representative images of VAV3 IHC staining from mouse liver. (J) Representative images of oil red O staining from mouse liver. (K-L) qPCR and Western blot showed the expressions of VAV3, ABCA1, Dhcr7 and Dhcr24. (M) Elisa result showed the serum level of IL-6. (N) qPCR showed mRNA levels of inflammatory cytokines IL-6, NF-kB, CCL2 and IL-1b in mouse liver. All data represent the means ± SEM; One-way ANOVA.

A mouse MAFLD model was established and hepatic-specific overexpression of VAV3 was conducted under HFD in the AAV group using rAAV-TBG-VAV3 (Figure 6A). During the 12-week feeding period, random and fasting blood glucose levels were measured, which showed significant improvement in glucose metabolic disorders in the AAV group compared to those in the HFD group (Figure 6B-C). In addition, the OGTT and ITT results suggested that impaired glucostasis and insulin sensitivity in the HFD group were ameliorated by the overexpression of VAV3 under HFD (Figure 6D-E). Hepatic overexpression of VAV3 also could reduce the increased body weight and liver size induced by HFD, as shown by body weight recording, gross pictures, and weight/tibia length ratio (Supplemental Figure 6B, Figure 6F-G). In addition, hepatic ultrasound results confirmed fatty liver disease in the HFD group, as indicated by the blue arrows with strong light areas compared to that in the CON group, whereas the manifestation in the AAV groups was milder (Supplemental Figure 6C). The serum levels of TG, TC, and LDL were also decreased, while HDL level was elevated in the AAV group compared to that in the HFD group, indicating that VAV3 overexpression could ameliorate lipid metabolism in MAFLD (Figure 6H). Furthermore, Oil red O staining showed that fewer lipid droplets accumulated in the AAV group than in the HFD group, while IHC staining verified hepatic-specific overexpression of VAV3 in the AAV group (Figure 6I-J, Supplemental Figure 6D). According to the results of qPCR and western blotting, VAV3 overexpression under HFD in the AAV group alleviated the intracellular accumulation of cholesterol *in vivo* compared to that in the HFD group (Figure 6K-L). The ELISA results also indicated a lower serum level of IL-6 in the AAV group than in the HFD group, and qPCR suggested that it could suppress many inflammatory cytokines in hepatic tissues by restoring VAV3 expression under HFD (Figure 6M-N). Overall, restoring the expression of VAV3 in the liver was proved to not only reduce glucolipid abnormalities but also suppress hepatic inflammation, eventually ameliorate the development of MAFLD.

## Materials and Methods

### Human liver cell lines

Human liver cancer cell lines (L02, HepG2) were purchased form the American Type Culture Collection (ATCC; Manassas, VA, USA) and cultured in Dulbecco's modified Eagle's medium (DMEM) (KeyGEN BioTECH, China) containing 22.5mM glucose supplemented with 10% fetal bovine serum (FBS; Hyclone Laboratories, USA) and 1% penicillin/streptomycin. All cell lines were cultured in a humidified 37˚C incubator with 95% air and 5% CO2.

### HFD-induced MAFLD mouse model

1. All animal studies were conducted in accordance with the standard procedures approved by the Institutional Animal Care and Use Committee of Tongji Hospital, Huazhong University of Science and Technology. 6-8 weeks of age, 22-25 g ApoE-/- mice were purchased from Charles River (Beijing, China). After adaptive feeding for 1 week, the mice were then randomized into two groups: i) control group (CON) (n=6): normal diet feeding; ii) HFD group (HFD)(n=6): HFD feeding. The mice were sacrificed after 8 weeks of treatment.

2. 6-8 weeks of age, 22-25 g ApoE-/- mice were purchased from Charles River (Beijing, China). After adaptive feeding for 1 week, the mice were then randomized into three groups: i) control group (CON)(n=6): vehicle AAV tail vein injection with normal diet; ii) HFD group (HFD)(n=6): vehicle AAV tail vein injection with HFD and iii) AAV group (AAV)(n=6): rAAV8-TBG-VAV3 tail vein injection with HFD. Weight and blood glucose were measured every week. The mice were sacrificed after 12 weeks of treatment.

### Mouse primary hepatocyte extraction

All the primary liver cells were extracted from ApoE-/- mice. Midline laparotomy was conducted after anesthesia. The hepatic portal vein and IVC were identified and stripped. Retrograde perfusion of the liver was conducted via the hepatic portal vein. The IVC was transected to allow outflow of perfusate. The liver was sequentially perfused with the following solutions at a flow rate of 5 mL/min (the perfusion apparatus, Longer Pump, China), firstly with 40 ml of Hanks' Balanced Salt Solution (without Ca2+ and Mg2+), then followed by 25ml of 0.01% collagenase Type IV dissolved in Hanks' Balanced Salt Solution (with Ca2+ and Mg2+). The temperature of all solutions was kept at 37°C. Lobes of the liver were collected and transferred into culture dish containing DMEM with 10% Penicillin-Streptomycin at 4°C for an interim. After that the hepatic tissues were transferred into PBS at 4°C and gently rinsed into the dispersed hepatocytes. The hepatocyte slurry was filtrated into a 50 ml centrifuge tube and was centrifuged at 200rpm for 2 minutes. The hepatocyte sediment was gently resuspended in 15 ml DMEM at room temperature. Then cells were then seeded with DMEM plating media containing 22.5 mM glucose, 10% fetal calf serum, 1% penicillin/streptomycin 30. The extracted primary hepatocytes were captured by microscope under 40 times as shown in the Supplemental Figure 1A, and we used trypan blue dye to detect the cell viability.

### Compounds

Oxidative-LDL (ox-LDL) was from Yiyuan (Beijing, China); it was stored at 4˚C before use and its concentration for storage was 2mg/mL. It was dissolved in DMEM to get 100 µg/mL concentration for cell experiments. IL-6 was from Cell Signaling Technology (Danvers, USA). It was dissolved in sterile water to get 25 µg/mL concentration for cell experiment, and was stored at -80˚ C. Stattic was purchased from Med Chem Express (Houston, USA). It was dissolved in sterile dimethyl sulfoxide (DMSO) to get 10 mM concentration for cell experiments, and was stored at -80 ˚C.

### Western blot analysis

Human liver cells were pretreated with stattic (10 µM) or DMSO for 2h. Then, cells were cultivated with ox-LDL (100 µg/mL) or vehicle for 24 h. Or cells were incubated with IL-6 (25 ng/mL) for 2, 6, 12 hours. After the treatments, cells were collected and washed with cold PBS and lysed on ice in a modified RIPA buffer (1% Triton X-100, 1% deoxycholate, 0.1% SDS) containing protease inhibitors (1 mM PMSF), subjected to SDS-PAGE. Proteins were transferred onto PVDF membrane and probed with antibodies purchased from Cell Signaling Technology company. Membranes were probed with a 1:1,000 dilution of primary antibodies against phospho-specific STAT3 (Cell Signaling Technology, Tyrosine 705, #9131), phosphor-independent STAT3 (Cell Signaling Technology, #4904), phospho-independent Akt (Cell Signaling Technology, #4685), phospho-specific Akt (Cell Signaling Technology, #4060), phospho-independent IR (Cell Signaling Technology, #23413), phospho-specific IR (Cell Signaling Technology, #3021), phospho-independent IRS (Cell Signaling Technology, #95816), phospho-specific IRS (Sigma-Aldrich, ZRB09432), VAV3 (Cell Signaling Technology, #2398s), GLUT4 (Cell Signaling Technology, #2213), Dhcr7 (ABclonal, A8049), Dhcr24 (ABclonal, A5402), ABCA1 (ABclonal, A7228) and GAPDH (Cell Signaling Technology, #2118). HRP-conjugated secondary antibodies were from Promotor Biotechnology Ltd. The specific proteins were detected using an enhanced chemiluminescence (ECL) Western blot kit according to the manufacturer's instructions.

### Immunohistochemical (IHC) staining

Formalin-fixed paraffin-embedded sections from mouse hepatic tissues were cut at 3-5 μm. Immunohistochemical staining was performed using monoclonal antibodies against phospho-specific STAT3 (1:500, Cell Signaling Technology, Tyrosine 705, #9131) and VAV3 (1:300, Cell Signaling Technology, #2398s). The histological staining was captured by microscope under 40 times and were compared and analyzed by GraphPad 8.0.

### Oil red O staining

Liver tissues were frozen and were stained with oil red O to analyze hepatic lipid droplets. Human liver cell lines were washed with PBS and fixed with 10% polyoxymethylene solution for 30min, and then were stained with oil red O for another 20 min. After that cells were washed with 60% isopropanol and PBS in turn for 3 times. At last, cell nuclei were stained with hematoxylin.

### Lipid metabolic test

The levels of TG, TC, LDL and HDL in mouse serum were evaluated by kits according to their protocol (Servicebio, China).

### Enzyme-linked immunosorbent assay (ELISA)

Secreted IL-6 level from L02 cells and mouse hepatic tissues were measured by ELISA kits (Servicebio, China), according to the manufacturer instructions.

### Chromatin immunoprecipitation sequencing (ChIP-seq) analysis

Chromatin immunoprecipitation assays were performed by Wuhan IGENEBOOK Biotechnology Co.,Ltd (http://www.igenebook.com) . Briefly, samples were washed twice in cold PBS buffer and cross-linked with 1% formaldehyde for 10 minutes at room temperature and then quenched by addition of glycine (125 mmol/L final concentration). Afterwards, samples were lysed and chromatins were obtained on ice. Chromatins were sonicated to get soluble sheared chromatin (average DNA length of 200-500 bp). 20ul chromatin was saved at -20°C for input DNA, and 100ul chromatin was used for immunoprecipitation by anti-STAT3 antibodies (Cell Signaling Technology, Tyrosine 705, #9131). 10 μg of antibody was used in the immunoprecipitation reactions at 4 °C overnight. The next day, 30 μL of protein beads was added and the samples were further incubated for 3 h. The beads were next washed once with 20 mM Tris/HCL (pH 8.1), 50 mM NaCl, 2 mM EDTA, 1% Triton X-100, 0.1% SDS; twice with 10 mM Tris/HCL (pH 8.1), 250 mM LiCl, 1 mM EDTA, 1% NP-40, 1% deoxycholic acid; and twice with TE buffer 1× (10 mM Tris-Cl at pH 7.5. 1 mM EDTA). Bound material was then eluted from the beads in 300 μL of elution buffer (100 mM NaHCO3, 1% SDS), treated first with RNase A (final concentration 8 μg/mL) during 6h at 65°C and then with proteinase K (final concentration 345 μg/mL) overnight at 45°C. Immunoprecipitated DNA was used to construct sequencing libraries following the protocol provided by the I NEXTFLEX® ChIP-Seq Library Prep Kit for Illumina® Sequencing (NOVA-5143-02, Bioo Scientific) and sequenced on Illumina with PE 150 method. Next, Trimmomatic (version 0.38) was used to filter out low-quality reads. Clean reads were mapped to the mouse genome by Bwa (version 0.7.15). Samtools (version 1.3.1) was used to remove potential PCR duplicates. MACS2 software (version 2.1.1.20160309) was used to call peaks by default parameters (bandwidth,300 bp; model fold, 5, 50; q value, 0.05). If the summit of a peak located closest to the TSS of one gene, the peak will be assigned to that gene. HOMER (version3) was used to predict motif occurrence within peaks with default settings for a maximum motif length of 12 base pairs. GO enrichment analysis was performed using the EasyGO gene ontology enrichment analysis tool (http://bioinformatics.cau.edu.cn/easygo/;). The GO term enrichment was calculated using hypergeometric distribution with a P value cutoff of 0.01. P values obtained by Fisher's exact test were adjusted with FDR for multiple comparisons to detect overrepresented GO terms. To reveal potential roles of genes, clusterProfiler (http://www.bioconductor.org/packages/release/bioc/html/clusterProfiler.html) in R package was employed to perform KEGG (Kyoto Encyclopedia of Genes and Genomes, http://www.genome.jp/kegg/) enrichment analysis.

### RNA interference

Small interfering RNA (siRNA)-to knock down STAT3 (si-STAT3), VAV3 siRNA (si-VAV3) and negative control NC (si-NC) were designed and synthesized by RiboBio (Guangzhou, China). L02 cell lines were transfected with the siRNAs using Lipofectamine 2000 (Thermo Fisher, USA). Cells were seeded in complete medium approximately 12h before transfection. Then, siRNAs mixed with Lipofectamine 2000 were added to the cells with fresh Opti-MEM medium (gibco, USA). SiRNAs were transfected at a concentration of 50 nM. After 6 h, the medium containing siRNAs and Lipofectamine 2000 was replaced with complete medium.

### shRNA

Lentivirus shRNA-VAV3 construct and non-silencing control construct were designed and provided by Genechem (Shanghai, China). 2x10^5 L02 cells were placed into each well of 6-well plate and were cultured overnight. Cells were transfected with the shRNA by using transfection P reagent and cultured for 36h before RNA and protein extraction. Transfected cells could be screened out by Puromycin.

### Plasmid transfection

STAT3-expression construct (NM_139276) and pENTER empty vector construct were designed and provided by Genechem (Shanghai, China). VAV3-expression construct (NM_006113) and pENTER empty vector construct were designed and provided by Vegene (Shandong, China). 2x10^5 L02 cells were placed into each well of 6-well plate and were cultured overnight. Cells were transfected with the plasmid using Lipo3000 and P3000 and cultured for 36h before RNA and protein extraction. Expression was detected via using qPCR and Western blot.

### RNA extraction and quantitative qRT-PCR (qPCR) analysis

Total RNA was purified by RNA purification kit (Magen, China) and reverse-transcribed using the ReverTra Ace® qPCR RT kit (TOYOBO, Japan) according to the manufacturer's instructions. SYBR® Green Realtime PCR Master Mix (TOYOBO, Japan) was applied to determining the mRNA levels of different genes using the primers listed in Supplemental Table 1B and GAPDH was used as an endogenous control.

### Cytosol and membrane separation assay

Membrane and cytosol protein extraction kit was purchased from Beyotime Bioengineering Institute (Shanghai, China). Membrane and cytosol proteins were extracted according to the manufacturer's instructions.

### 2-DG glucose uptake analysis

Cellular glucose uptake was analyzed via using the fluorescent glucose analog, 2-DG with slight modification. Briefly, cells were plated in 96-well plates (1 × 104 cells per well). After the treatments, 2-DG was added with a final concentration of 100 µg/mL for 2h at 37 °C. Then, the reaction was terminated by adding stop buffer and neutralization buffer. Then cells were washed twice with PBS and were added with serum-free medium. Fluorescence intensity was immediately measured in a micro-plate reader at an excitation wavelength of 485 nm and an emission wavelength of 530 nm. A fair estimation of the overall glucose uptake was obtained by quantifying the fluorescence.

### Immunofluorescence staining

Cells were seeded on sterile glass slides and grew overnight. L02 cells were treated with ox-LDL and IL-6 for 24h or 6h respectively. Before fixed by methanol, cells in insulin groups were stimulated by insulin at the concentration of 100ug/mL. Then they were washed with ice-cold PBS buffer, and were fixed with ice-cold methanol at room temperature for 15 min. After three times washing with PBS buffer, cells were blocked with 0.5% normal goat serum (dissolved in PBS buffer) at room temperature for 1 h. Then cells were probed with rabbit antibody to GLUT4 (1:100 dilution) at 4˚C overnight. After the overnight incubation, cells were washed with PBS buffer containing 0.1% Tween-20, and then were incubated with Cy3-conjugated anti-rabbit secondary antibody (1:200; Jackson ImmunoResearch Laboratories, West Grove, USA) at room temperature for 1 h. Later the cells were incubated with WGA-FITC (Sigma, USA), a specific membrane label, at the concentration of 5µg/mL for another 10min. Then cells were incubated for 5 min at room temperature with DAPI (vector Laboratories, Burlingame, USA) to stain nuclei and were observed by using an inverted fluorescence microscope.

### AAV tail vein injection

rAAV8-TBG-VAV3 was structed by Vegene company (Shandong, China) aiming to overexpression VAV3 in liver specifically. Mice were injected with rAAV8-TBG-VAV3 at a final concentration of 2^1011genome copies per mL in AAV group while mice in other 2 groups were injected with vehicle AAV. Two weeks after injection VAV3 should be overexpressed in liver. We have conducted preliminary experiment to detect the specific expression efficiency of this rAAV8-TBG-VAV3 by immunofluorescence as shown in Supplemental Figure 5A.

### Statistical analysis

The data were presented as the mean ± SEM for at least three independent experiments. Statistical analysis was performed with GraphPad 8.0. The significant differences between any of the two groups were evaluated by one-way analysis of ANOVA or AUC analysis. If only two groups, they were evaluated by t-test analysis. Statistical significance was defined as P<0.05.

## Discussion

Metabolic-associated fatty liver disease (MAFLD), once called non-alcoholic fatty liver disease (NAFLD), is the most prevalent chronic hepatic disease in the world 31, 32. According to abundant clinical evidence, MAFLD is considered an independent risk factor for many severe chronic diseases, such as chronic kidney disease, T2DM, and cardiovascular diseases, causing systemic metabolic disorder and inflammatory infiltration 7, 33. Hence, early intervention for MAFLD is meaningful to delay the development of multiple serious chronic diseases.

Based on previous studies, there is no unified method for establishing MAFLD mouse models 34, 35. In many studies, ApoE^-/-^ mice were fed with HFD at approximately 8-12 weeks 36, 37. All MAFLD mouse models were testified by Oil red O staining and other metabolic-associated tests to prove successful modeling. In our study, ApoE^-/-^ mice were fed with HFD for 8 weeks to elicit the early phase of MAFLD, and were verified by Oil red O staining and metabolic blood tests 38. A significant increase in the level of fasting and random blood glucose was then observed in mice from the HFD group compared to those in the CON group. Furthermore, we conducted OGTT/ITT tests to verify glucose abnormalities and insulin inertia in the HFD-fed mice. According to previous studies, extracellular glucose is absorbed into hepatocytes through GLUT4, which is mediated by insulin, and translocated into the membrane 13, 39. GLUT4 is usually stored in GSVs in the cytosol. Once insulin is secreted by the pancreatic islets and sensed by hepatocytes, the canonical insulin signaling pathway IRS/IR/AKT is activated 40. GLUT4 then rapidly responds to the recruitment of insulin and is transported into the membrane for glucose uptake 14, 15. The canonical insulin signaling pathway IRS/IR/AKT and GLUT4 total expression remained unchanged in the 8-week mouse model, regarded as the early phase of MAFLD. This suggested that GLUT4 membrane translocation might be the main cause of abnormal glucose absorbance during this phase. Nevertheless, based on the results of cytosol-membrane extraction in primary hepatocytes, a deficiency in GLUT4 membrane traffic was observed, supporting our hypothesis. To further explore the specific mechanism of abnormal GLUT4 translocation in MAFLD, the study was focused on HFD-induced hepatic inflammation as it was previously reported that chronical low level of inflammation could promote metabolic disorders 4, 41. Multiple inflammatory cytokines were activated in the livers of the HFD group, and focus was placed on elevating IL-6 levels. This is because the IL-6/STAT3 signaling pathway is activated in many chronic inflammatory diseases, such as cardiovascular disease and cancer. Once IL-6 combines with an IL-6 receptor (IL-6R), it forms a tripolymer with the co-receptor glycoprotein 130 and triggers cellular STAT3 to phosphorylate and translocate into the nucleus via dimerization of P-STAT3. This acts as a transcription factor to modulate gene expressions 10. Additionally, IL-6 was adopted to activate STAT3, which caused a deficiency in GLUT4 membrane traffic and abnormal glucose uptake in L02 cells, emphasizing the need to consider the association between STAT3 and abnormal GLUT4 membrane translocation in MAFLD.

In this study, significant hepatic STAT3 activation was observed both in the HFD group or after stimulated by ox-LDL. Considering that activated STAT3 can act as a transcriptional factor, ChIP-seq analysis was conducted to explore the association between STAT3 and GLUT4, and the specific regulatory mechanism against glucose uptake in MAFLD. However, according to the ChIP-seq results, STAT3 could not directly regulate GLUT4 expression by binding to its promoter region. Based on GO and KEGG analyses, STAT3 might transcriptionally regulate the expression of VAV3, in accordance with predictions in the JASPAR database. As a GEF to activate Rho GTPase, VAV3 participates in multiple biological processes, such as endothelial defensive function, cancer metastasis, and even glucose homeostasis via regulation of GSV directional traffic 18, 29, 42, by switching non-activated GDP-binding GTPases to activated GTP-binding GTPases, such as Rho A, Rac-1, and CDC42 17, 18, 29. It has also been reported to have latent susceptibility to hypertension, diabetes, and obesity and is associated with the risk of elevated basic blood glucose 43-45. According to current research, the directional traffic of GSVs from the cytosol to the membrane in response to insulin is partly dependent on GTPases for offering anchors and motors, indicating that VAV3 might regulate GLUT4 membrane translocation 46, 47. In addition, shVAV3-L02 cell lines were constructed via lentivirus to consistently knockdown VAV3 and demonstrated VAV3 defect induced deficiency in GLUT4 membrane translocation and impaired glucose absorbance. Moreover, our results indicated that HFD induced STAT3 activation and decreased the expression of VAV3 both *in vitro* and *in vivo*. STAT3 overexpression could decrease VAV3 expression, whereas inhibiting STAT3 could elevate the expression of VAV3, verifying the negative regulation between STAT3 and VAV3.

Since glucose and lipid metabolism can interact with each other in metabolic disorders 23, studies should not only concentrate on disturbed glucose metabolism but also focus on whether there are lipid metabolic abnormalities in MAFLD. According to our results, ox-LDL stimulation increased intracellular cholesterol synthesis with elevated cholesterol synthetases Dhcr7, Dhcr24, and Cyp51, but reduced cholesterol efflux with decreased reverse cholesterol transporter ABCA1, accelerating hepatic intracellular cholesterol accumulation. Previous studies have indicated that excessive cholesterol accelerates hepatic steatosis, modulates inflammation, and induces MAFLD progression 48, 49. In addition, STAT3/VAV3 was verified to affect hepatic cholesterol accumulation based on the finding that recovering the expression of VAV3 under ox-LDL stimulation or suppression of STAT3 activation could attenuate cholesterol disorders. Considering the fact that GLUT4 translocation was regulated by STAT3/ VAV3 axis, the improved glucose intracellular uptake might indirectly influence the condition of cholesterol metabolism. However, the specific mechanism of VAV3 deficiency inducing intracellular cholesterol accumulation requires further exploration. Apart from glucolipid interactions in metabolism, there might be more direct explanations for the role of VAV3 in cholesterol metabolism.

Moreover, to determine whether VAV3 could be a latent therapeutic target during the development of MAFLD *in vivo*, VAV3 was overexpressed specifically in the mouse liver under HFD feeding via rAAV8-TBG-VAV3. Restoring VAV3 expression significantly attenuated the impaired glucolipid disorders which induced by HFD and delayed the progression of MAFLD to a certain degree. Moreover, the increased hepatic inflammatory cytokines induced by HFD was ameliorated by restoring VAV3 expression. The alleviated chronic inflammatory status might be on account of improved glucolipid metabolism. In our study, STAT3 activation induced by HFD was observed during MAFLD development, causing decreased expression of VAV3. Interestingly, VAV3 defect could result in glucolipid metabolic disorders, promoting further activation of STAT3, probably due to the deterioration of MAFLD. However, recovering the expression of VAV3 reduced by HFD might correct the entire adverse loop in MAFLD progression. Hyperactivated STAT3 was also suppressed when VAV3 was overexpressed in HFD mice (Supplementary Figure 6E).

**Figure 7:**
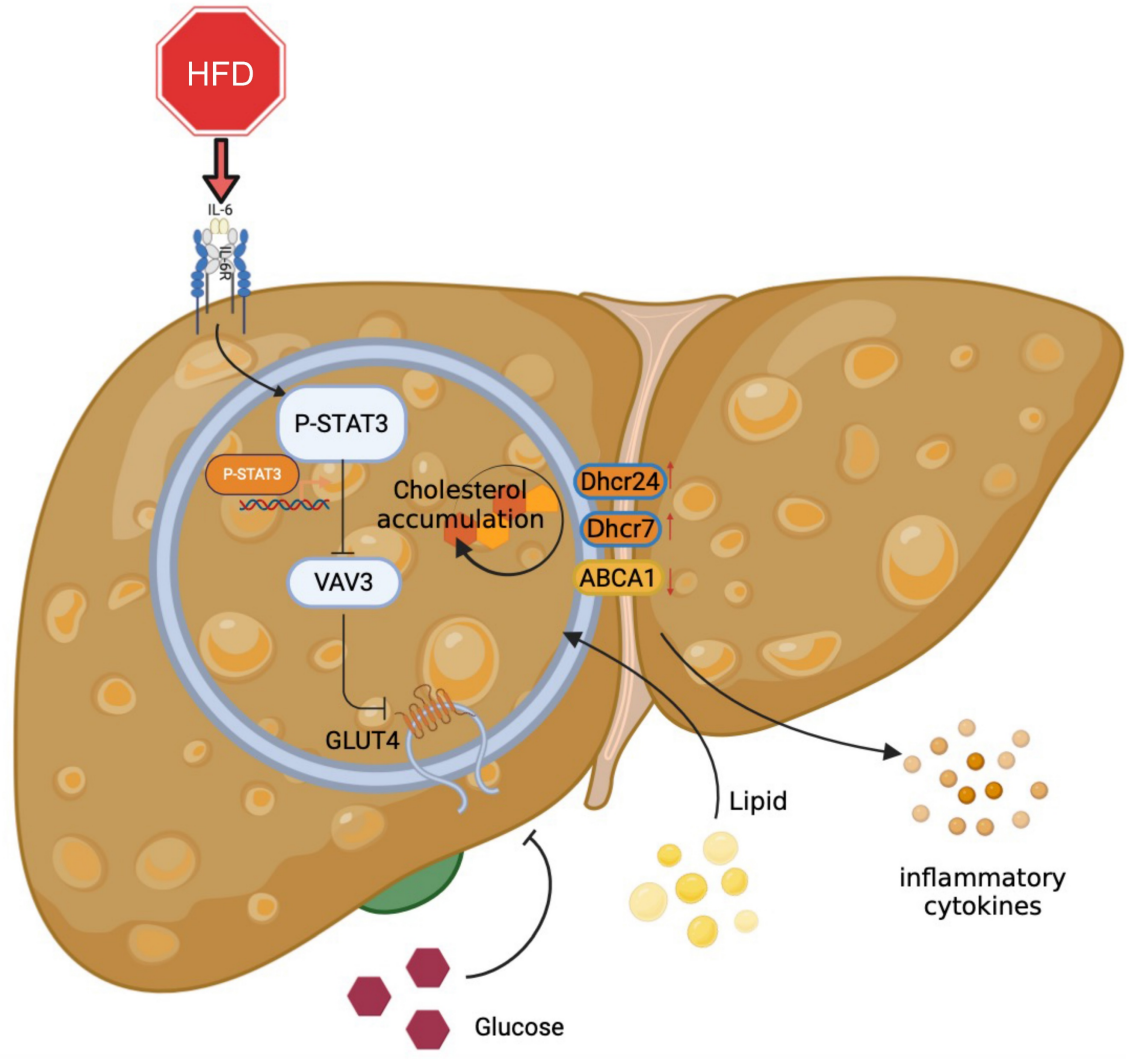
Graphical abstract explained the machinery of the development of MAFLD, which STAT3/VAV3 axis participated in. Figure was created with Biorender.

Overall, the major role of the STAT3/VAV3 axis in the development of MAFLD was elucidated, along with its potential to result in glucolipid metabolic dysfunction and acceleration of hepatic inflammation (Figure 7). Recovering the expression of VAV3 under HFD was regarded as a potential strategy for treating MAFLD, alleviating glucolipid metabolism, and relieving hepatic inflammation.

## Supplementary Material

Supplementary figures and tables.
