# Supplementary material for: The role of STAT3/VAV3 in glucolipid metabolism during the development of HFD-induced MAFLD
Source: Int J Biol Sci. 2024 Mar 11;20(6):2027–43. doi: 10.7150/ijbs.86465 (PMC11008271; doi:10.7150/ijbs.86465)
Supplement: Supplementary file 1 — Supplementary figures and tables. [file ijbsv20p2027s1.pdf]

Supplemental Figure 1

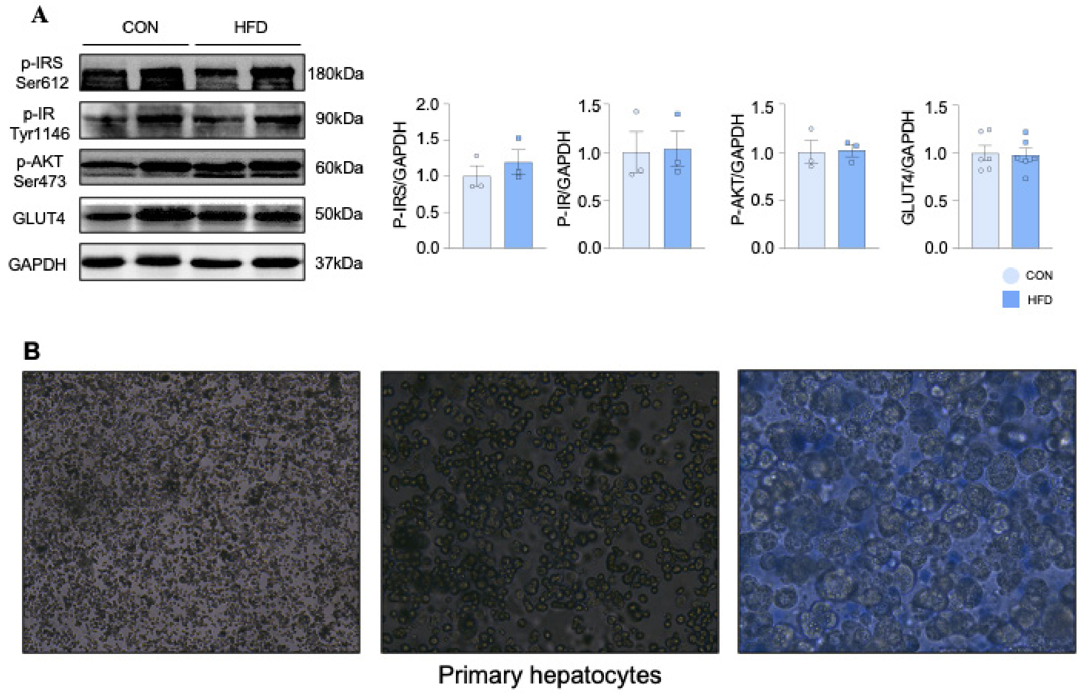

(A) Western blot showed expressions of P-IRS, P-IR, P-AKT, and GLUT4. (B) Representative images of the isolated primary hepatocytes with trypan blue dye to detect the cell viability.

Supplemental Figure 2

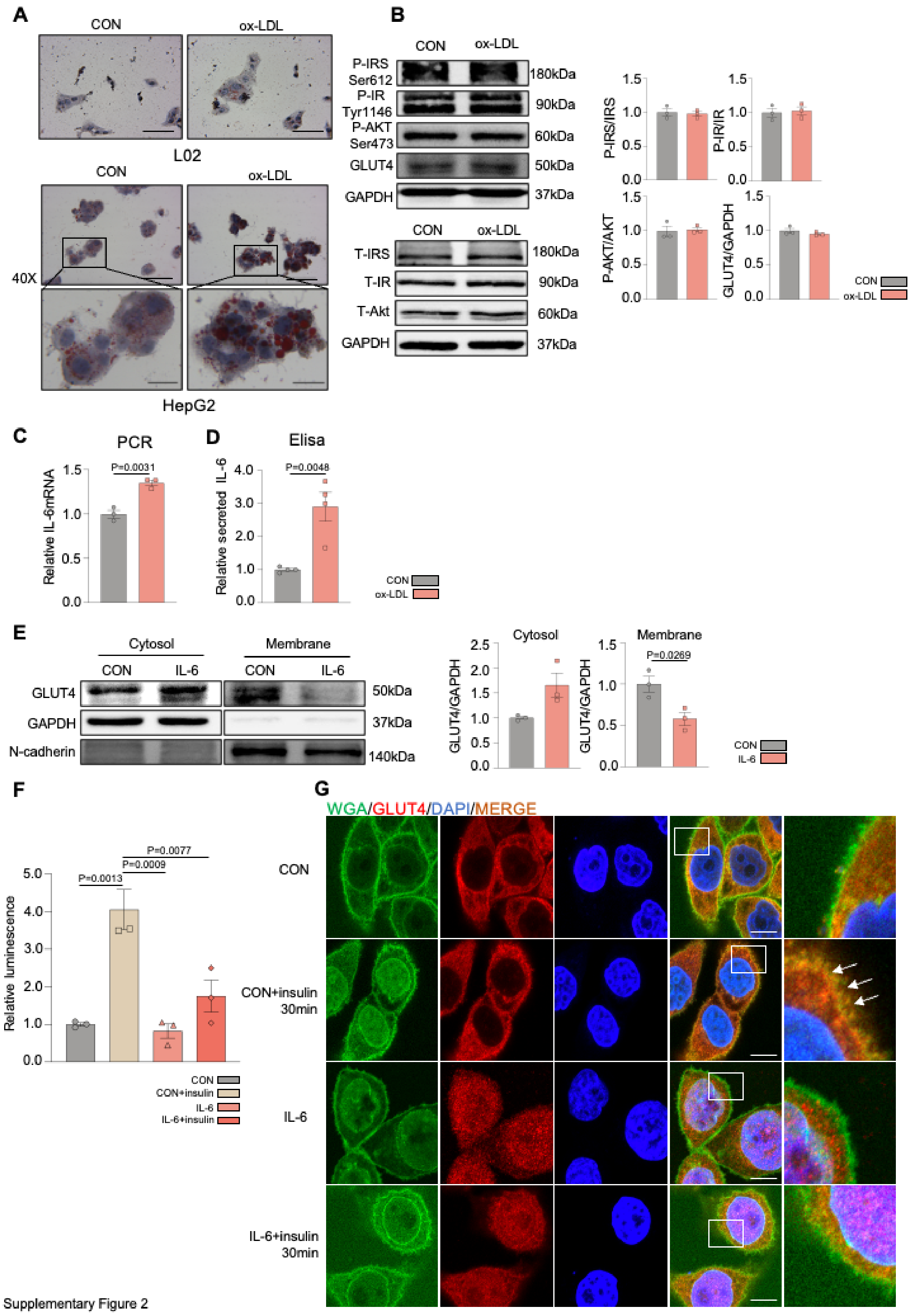

(A) Representative images of oil red O staining in L02 and HepG2 cells. (B) Western blot results demonstrated the expressions of P-STAT3, total IRS (T-IRS), total IR (T-IR), total AKT (T-AKT) and GLUT4 in L02 cells. (C) qPCR demonstrated IL-6 mRNA level in L02 cells. (D) ELISA result showed IL-6 level in L02 cell supernatant. (E)

Cytosol-membrane extracting demonstrated the GLUT4 distribution on membrane and cytosol in L02 cells under IL-6 stimulation. (F) Luminescence showed the glucose uptake level of L02 cells detected by 2-DG glucose uptake assay. (G) Representative images of immunofluorescence showed the colocation of membrane marker WGA (FITC-Green) and GLUT4 (Cy3-Red). Blue represents nuclear DNA staining by DAPI; green represents WGA staining; red represents GLUT4 staining. Data represent means ± SEM; t-test; One-way ANOVA.

Supplemental Figure 3

A

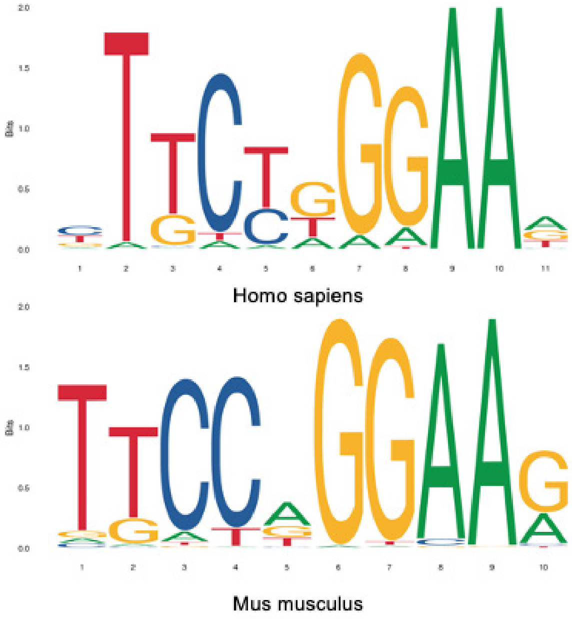

(A) Prediction biding sites of STAT3 in JASPAR database.

Supplemental Figure 4

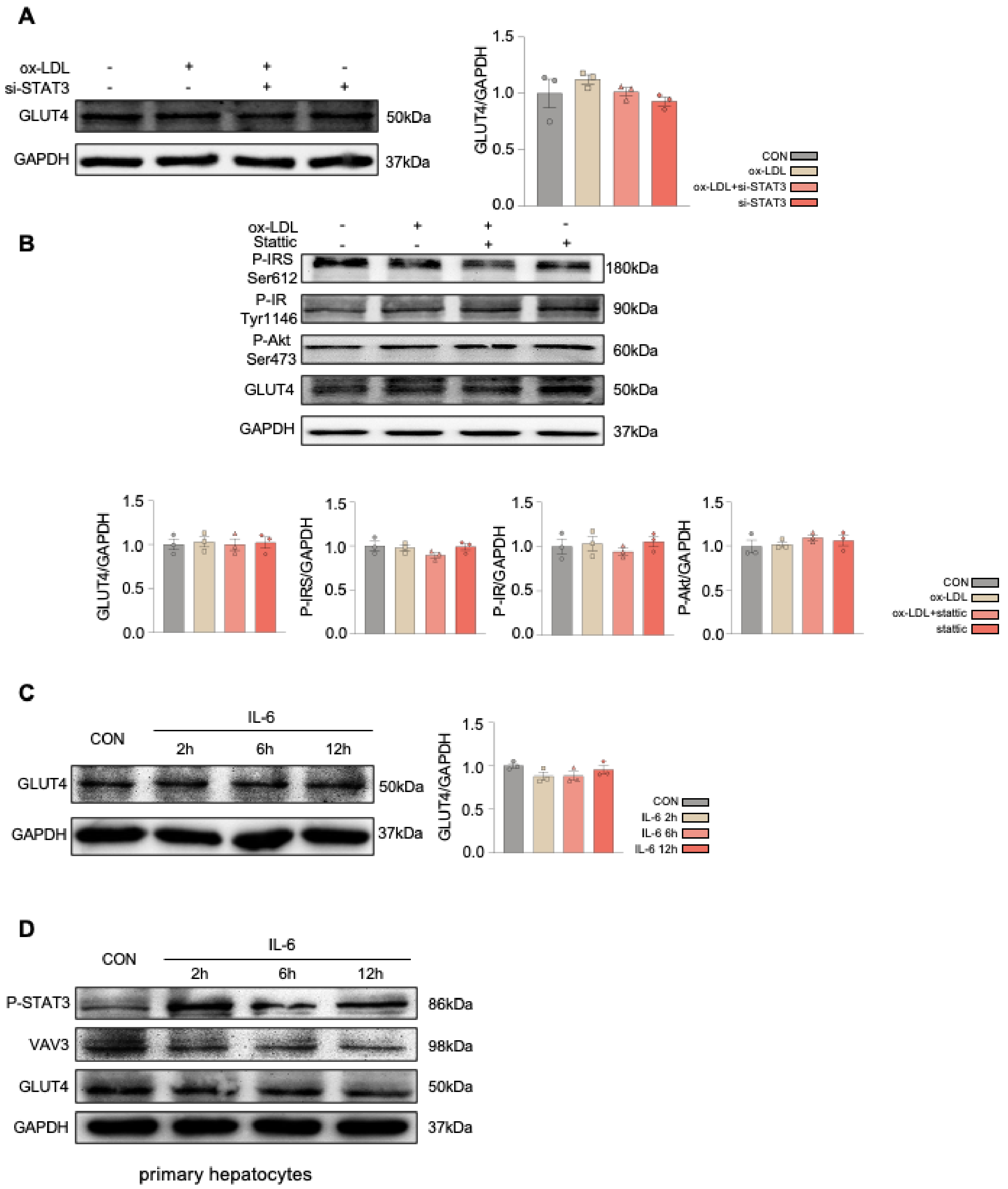

(A) Western blot results showed the expressions of GLUT4 in L02 cells under ox-LDL stimulation with si-STAT3 or stattic. (B) Western blot results showed the expressions of GLUT4, P-IRS, P-IR and P-Akt in L02 cells under ox-LDL stimulation with stattic. (C) Western blot results showed the expressions of GLUT4 in L02 cells under IL-6 time-dependent manner 2h, 6h and 12h. (D) Western blot results showed the expressions of P-STAT3, VAV3 and GLUT4 in isolated primary hepatocytes under IL-6 time-dependent manner. All data represent the means ± SEM; One-way ANOVA.

Supplemental Figure 5

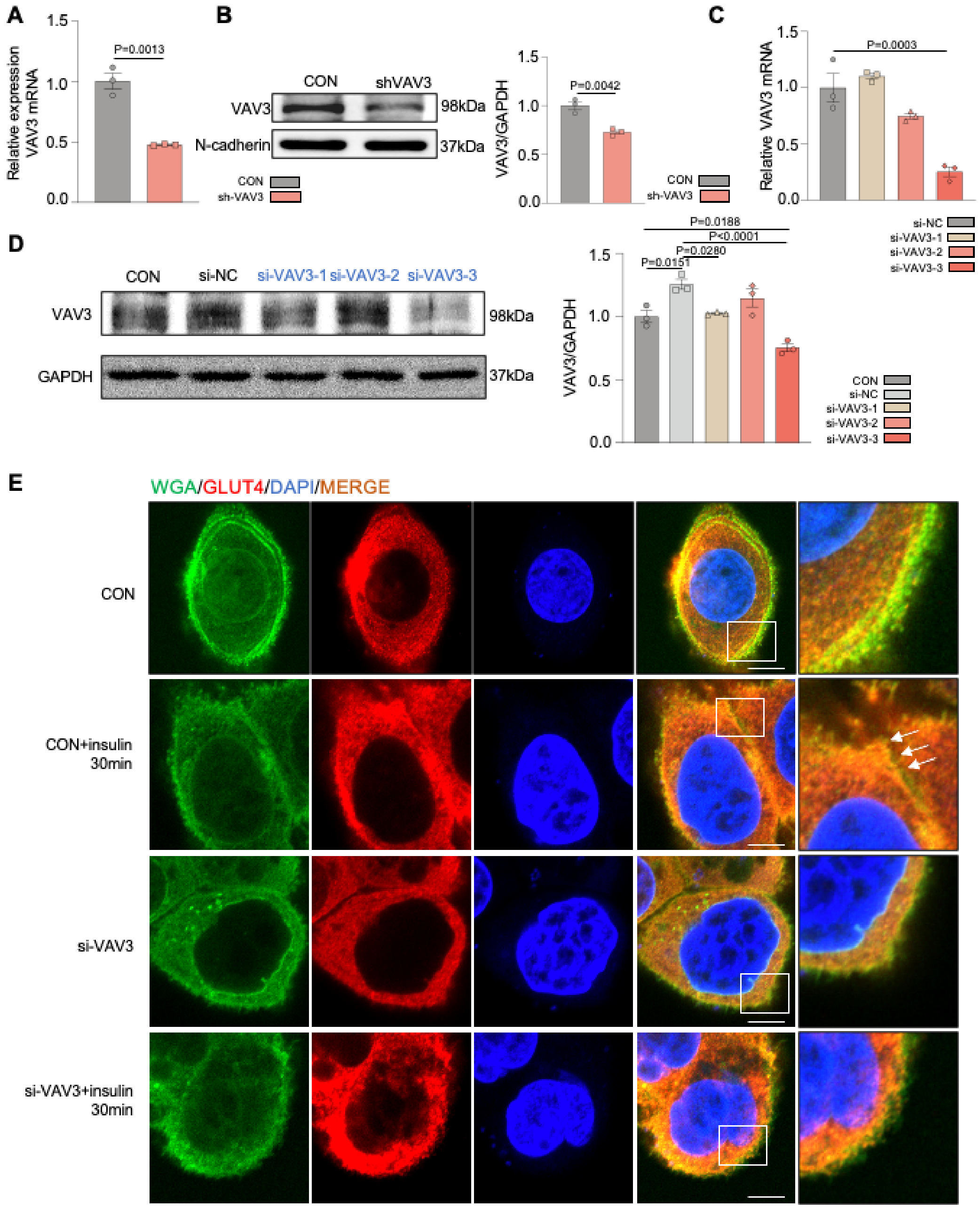

Supplementary Figure 5

(A-B) qPCR and Western blot results showed the knock-down efficiency of shVAV3. (C-D) qPCR and Western blot results showed the different knock-down efficiencies of si-VAV3. (E) Representative images of immunofluorescence showed the colocation of membrane marker WGA (FITC-Green) and GLUT4 (Cy3-Red). Blue represents

nuclear DNA staining by DAPI; green represents WGA staining; red represents GLUT4 staining. All data represent the means ± SEM; t-test; One-way ANOVA.

Supplemental Figure 6

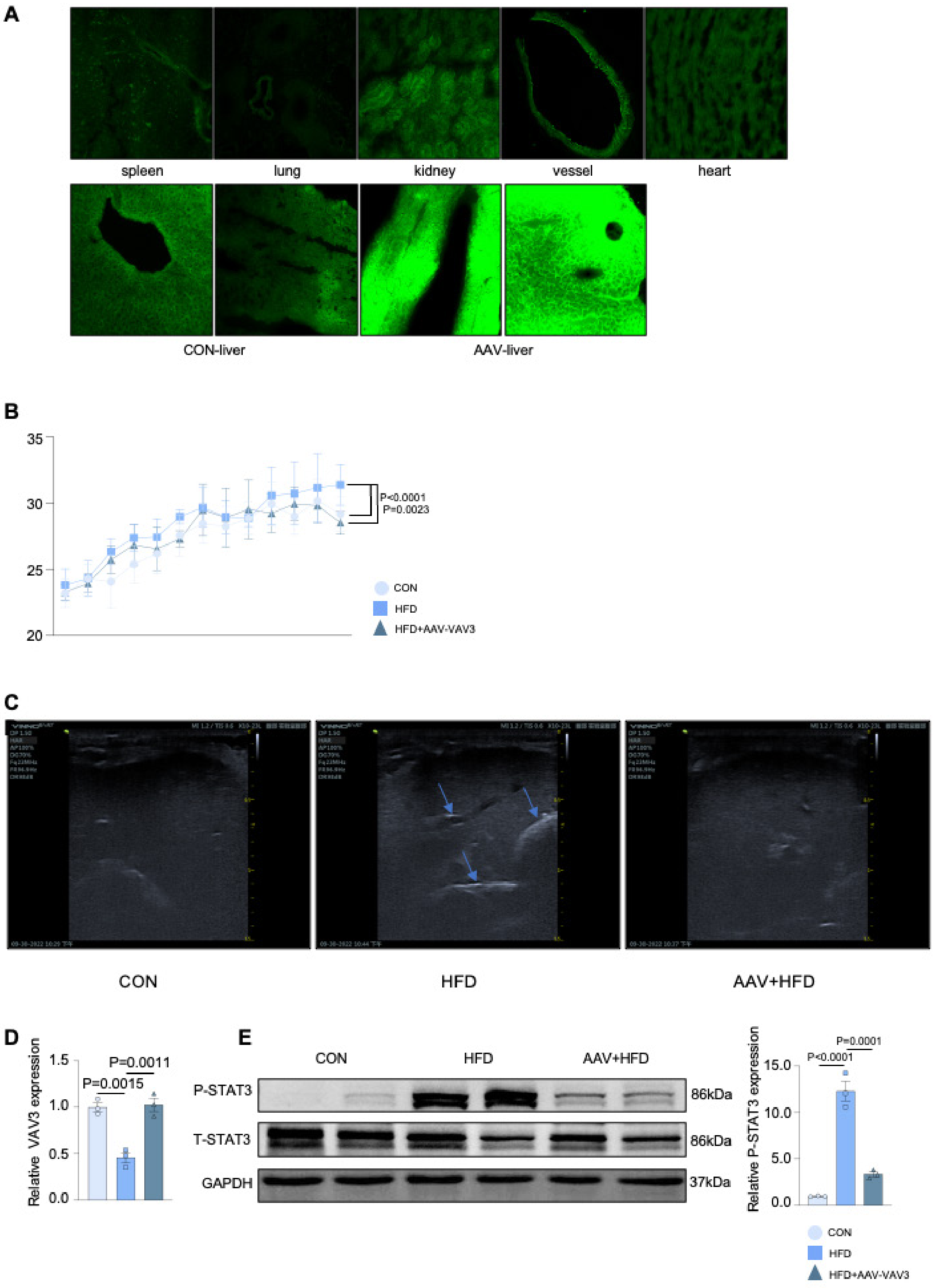

Supplementary Figure 6

(A) Representative images of immunofluorescence showed the rAAV8-TBG-VAV3

transfection efficiency in different organs after 2-week injection. (B) Statistic analysis demonstrated body weight variation in 3 groups. (C) Representative images of the hepatic ultrasound. (D) IHC staining results of VAV3 were plotted as histograms through statistical analysis. (E) Western blot results showed the expression of P-STAT3. All data represent the means ± SEM; One-way ANOVA.

Supplementary Table 1

(A) The prediction results of STAT3-bingding sites within VAV3 sequence region from JASPAR database.

Score	Start	End	Strand	Predicted site sequence
12.187638	1819	1828	+	TTTCAGGAAG
11.359834	784	793	+	TTCCAGAAAG
10.398615	106	115	+	TGTCAGGAAG
10.019197	1818	1827	-	TTCCTGAAAG

(B) The primers used in qPCR are listed.

Species	Gene	Forward primers	Reverse primers
Human	IL-6	GACAGCCACTCACCTCTTCAGAAC	GCCTCTTTGCTGCTTTCACACATG
Human	GAPDH	CAGGAGGCATTGCTGATGAT	GAAGGCTGGGGCTCATTT
Human	VAV3	ATTGCCATCGCTCGGTATGACTTC	GCCCACCCTGCCATTTACTTCTC
Human	ABCA1	TTTTTGCTCAGATTGTCTTGCC	TGTACTGTTCGTTGTACATCCA
Human	Cyp51	AGGCGATGGAGAAGGTGACAGG	GTAGACCAGGCTGAGGGTGAAGG
Human	Dhcr24	GAGTCATCATCCCACAAGTACG	TAGAACAGGTCTGAGTTTTCGG
Human	Dhcr7	TGATTGACTTCTTCTGGAACGA	TCATCTGCAGCGTGTAAAGATA
Mouse	IL-1b	CACTACAGGCTCCGAGATGAACAAC	TGTCGTTGCTTGGTTCTCCTTGTAC
Mouse	CCL2	TTTTTGTCACCAAGCTCAAGAG	TTCTGATCTCATTTGGTTCCGA
Mouse	NF-kB	CAAAGACAAAGAGGAAGTGCAA	GATGGAATGTAATCCCACCGTA
Mouse	Dhcr7	TGCTGCTTTATTCCTGGCTTCCTG	GCTGGAGTAATGGCACCTTCTTGG
Mouse	Dhcr24	CCCTGGTTCTTCAAGCATGTGGAG	TGTGTCGGTGGTAGTAGTGTCTCAG
Mouse	ABCA1	AGAAGGAGGCTCGGCTGAAGG	GAGGGATGAGGCTGCTAACAAACC
Mouse	STAT3	TGTCAGATCACATGGGCTAAAT	GGTCGATGATATTGTCTAGCCA
Mouse	IL-6	CTTCTTGGGACTGATGCTGGTGAC	TCTGTTGGGAGTGGTATCCTCTGTG
Mouse	GAPDH	GGTTGTCTCCTGCGACTTCA	TGGTCCAGGGTTTCTTACTCC

(A) The prediction results of STAT3-bingding sites within VAV3 sequence region from JASPAR database. (B) The primers used in qPCR were listed below.
